# Supplementary material for: Primary somatosensory cortex bidirectionally modulates sensory gain and nociceptive behavior in a layer-specific manner
Source: Nat Commun. 2023 May 24;14:2999. doi: 10.1038/s41467-023-38798-7 (PMC10209111; doi:10.1038/s41467-023-38798-7)
Supplement: Supplementary file 1 — Supplementary Information [file 41467_2023_38798_MOESM1_ESM.pdf]

Supplementary Materials

Primary Somatosensory Cortex Bidirectionally Modulates Sensory Gain and Nociceptive Behavior in a Layer-Specific Manner

Katharina Ziegler[1], Ross Folkard[1], Antonio J Gonzalez[1], Jan Burghardt[1], Sailaja Antharvedi-Goda[1], Jesus Martin-Cortecero[1], Emilio Isaías-Camacho[1], Sanjeev Kaushalya[2], Linette Liqi Tan[2], Thomas Kuner[3], Claudio Acuna[4], Rohini Kuner[2], Rebecca A Mease[1*] and Alexander Groh[1*]

1 Medical Biophysics, Institute for Physiology and Pathophysiology, Heidelberg University, Germany
2 Department of Molecular Pharmacology, Institute for Pharmacology, Heidelberg University, Germany
3 Institute for Anatomy and Cell Biology, Heidelberg University, Germany
4 Chica and Heinz Schaller Research Group, Institute for Anatomy and Cell Biology, Heidelberg University, Germany

These authors contributed equally: Katharina Ziegler, Ross Folkard, Antonio J Gonzalez.
These authors jointly supervised this work: Rebecca A Mease, Alexander Groh.
* Corresponding authors: Rebecca A Mease beckin@gmail.com, Alexander Groh groh@uni-heidelberg.de.

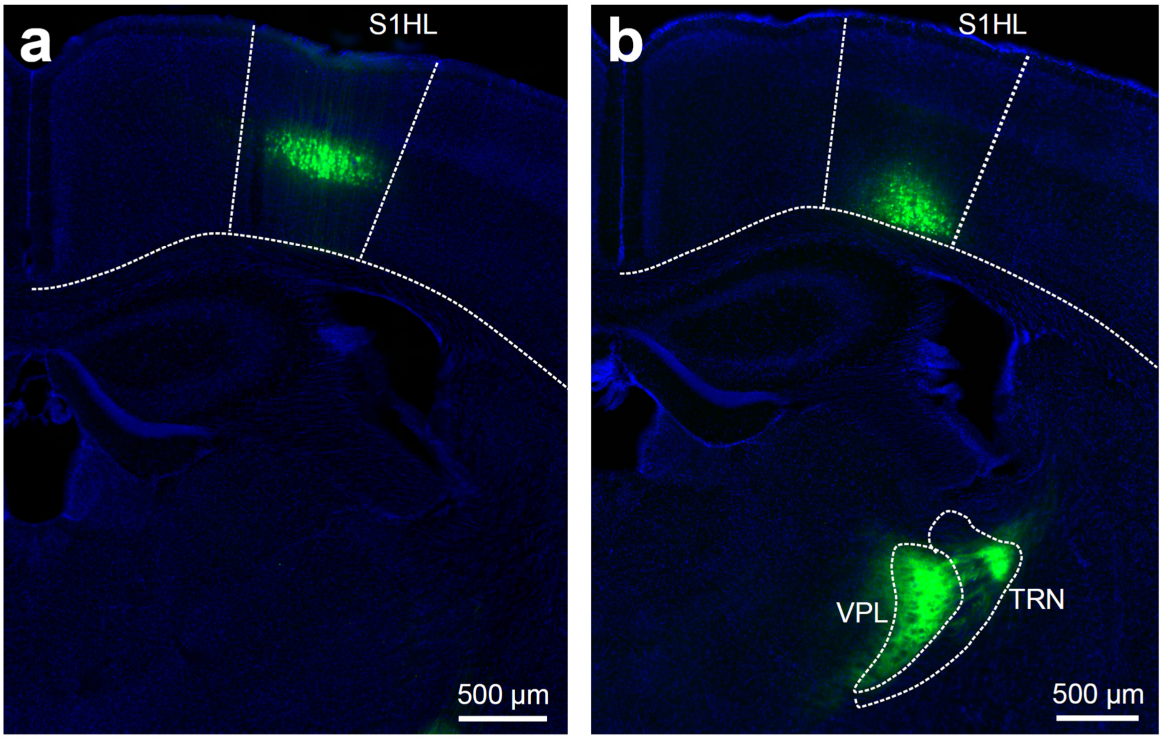

Supplementary Fig. 1: EGFP expression in control animals. Related to Figures 1-6.

(a) Cre-dependent expression of DIO-EGFP (green) in Layer 5 of the S1 hindlimb cortex (S1HL) of Rbp4-Cre mice. Representative example of *n* = 29 independent experiments with similar results. **(b)** Cre-dependent expression of DIO-EGFP (green) in Layer 6 of the S1HL cortex of Ntsr1-Cre mice. Slices were stained with DAPI (blue). Representative example of *n* = 23 independent experiments with similar results.

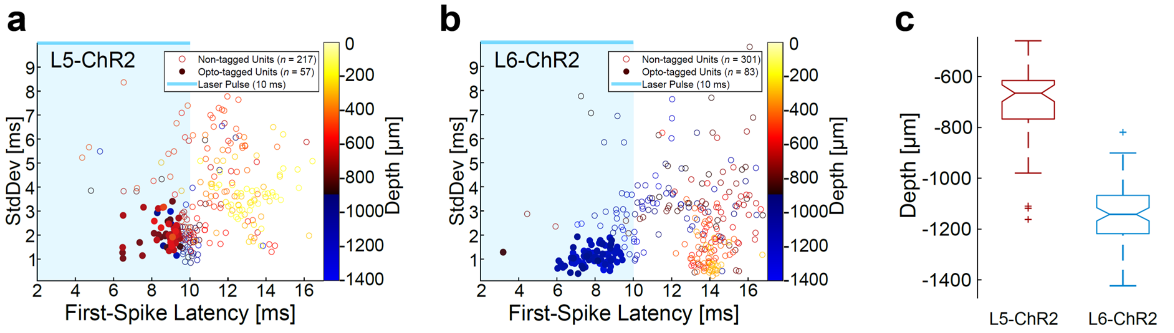

Supplementary Fig. 2: Optotagging of L5-ChR2 and L6-ChR2 Single Units.

10 Hz laser trains (10 ms pulse length) were given to Rbp4-Cre-ChR2-EYFP (*n* = 2 mice) or Ntsr1-Cre-ChR2-EYFP mice (*n* = 3 mice) as part of a 5 second on 5 second off protocol (> 1000 pulses in total per mouse). The mean first-spike latency and the standard deviation of the first spike latency to all 10 ms laser pulses was calculated for every single unit from a pooled dataset of each mouse line. Putative fast-spiking (FS) units (Δtrough-to-second-peak of extracellular mean waveform < 215 µs [1]) were removed from the tagged populations.
(a) Scatter plot of mean first spike latency and standard deviation of first spike latency in S1 hindlimb cortex (S1HL) of L5-ChR2 mice (*n* = 2). Tagged units (57/274 - filled circles) were assigned by mean latency < 9.5 ms and standard deviation < 3.5 ms. **(b)** Scatter plot of mean first spike latency and standard deviation of first spike latency in S1HL of L6-ChR2 mice (*n* = 3). Tagged units (83/384 - filled circles) were assigned by mean latency < 9.5 ms and standard deviation < 2 ms. **(c)** Box and Whisker plot of Opto-tagged unit depths for L5-ChR2 and L6-ChR2 data in **(a)** and **(b)**. The median L5-ChR2 unit depth was -665.5 µm (IQR = 151.5 µm), and the median L6-ChR2 unit depth was -1157.5 µm (IQR = 150.75 µm). Source data for a-c are provided as a Source Data file.

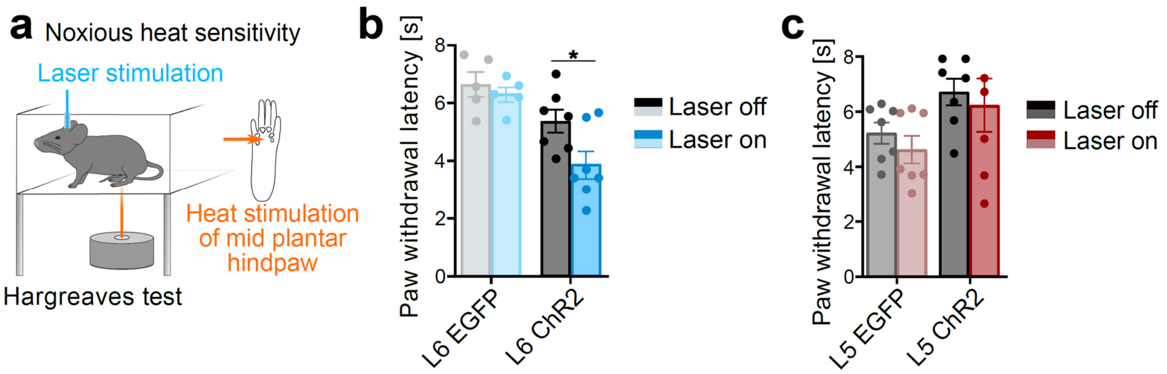

Supplementary Fig. 3. Hargreaves test in Layer 6 (L6) and Layer 5 (L5).

(a) Schematic of Hargreaves test [2] to quantify noxious heat sensitivity in response to noxious heat laser stimulation (orange) of the hindpaw with and without optogenetic stimulation in S1 hindlimb cortex (S1HL).

(b) Paw withdrawal latencies in response to heat stimulation of the left hindpaw with (blue, Laser on, 5 s continuous pulse) and without (black/gray, Laser off) optogenetic stimulation of contralateral L6 corticothalamic (L6-CT) neurons in S1HL of L6-EGFP (n = 5) and L6-ChR2 (n = 7) mice.

(c) Paw withdrawal latencies in response to heat stimulation of the left hindpaw with (Laser on, red, 5 s continuous pulse) and without (black, Laser off) optogenetic stimulation of L5 in the contralateral S1HL of L5-EGFP (n = 7) and L5-ChR2 (n = 7) mice.

* represent $p < 0.05$; b-c: two-way repeated measures ANOVA with post-hoc Bonferroni test. Exact F and p values in Supplementary Table 1. Data are shown as mean ± S.E.M. Source data for b-c are provided as a Source Data file.

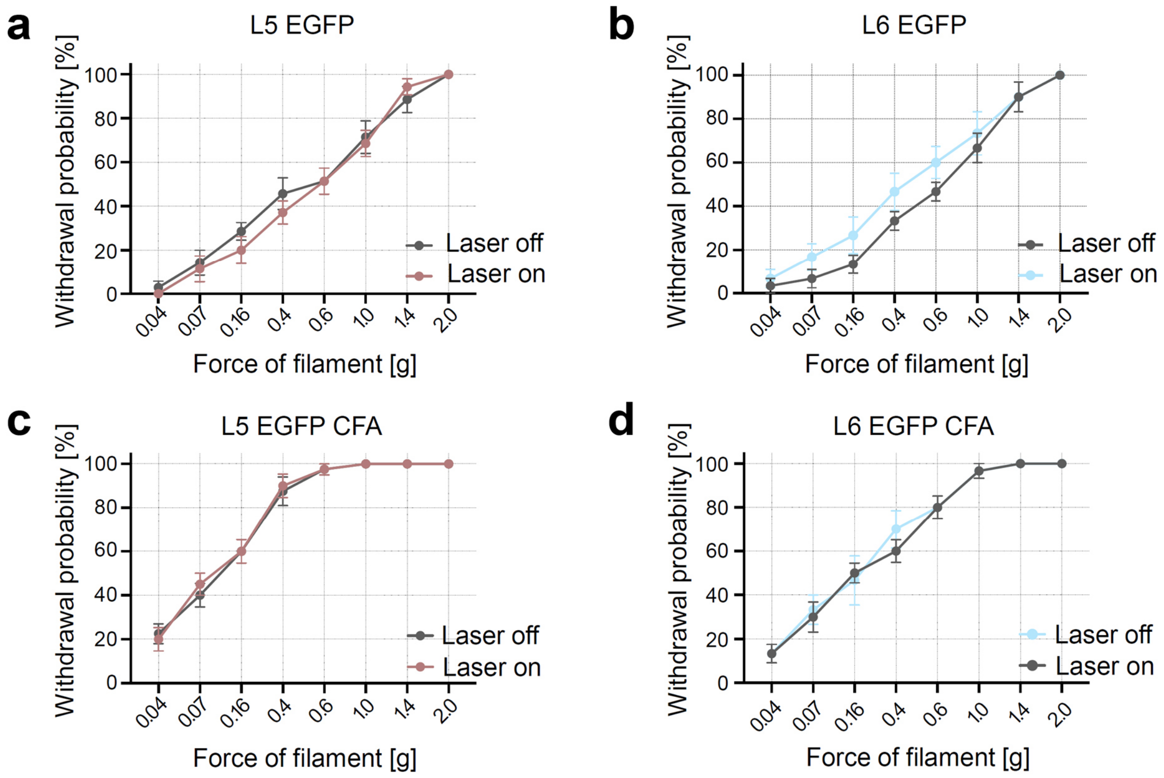

Supplementary Fig. 4: von Frey mechanical sensitivity test in EGFP control mice with and without optogenetic stimulation in S1 hindlimb cortex (S1HL) with a fiber implant. Related to Figures 2, 5 and 6.

Each panel shows averaged within-animal comparison of paw withdrawal probabilities in response to graded von Frey stimulation of the hindpaw at baseline (Laser off, black lines) and during laser stimulation (Laser on, 5 s continuous, red and blue lines) in the contralateral S1HL of Rbp4-Cre (red lines) and Ntsr1-Cre (blue lines) mice injected with AAV-DIO-EGFP. **(a)** L5-EGFP, n = 6 mice **(b)** L6-EGFP, n = 6 mice **(c)** L5-EGFP, Complete Freund's adjuvant (CFA), n = 8 mice **(d)** L6-EGFP, CFA, n = 6 mice.

Two-way repeated measures ANOVA with post-hoc Bonferroni test. Exact F and p values in Supplementary Table 1. Data are shown as mean ± S.E.M. Source data for a-d are provided as a Source Data file.

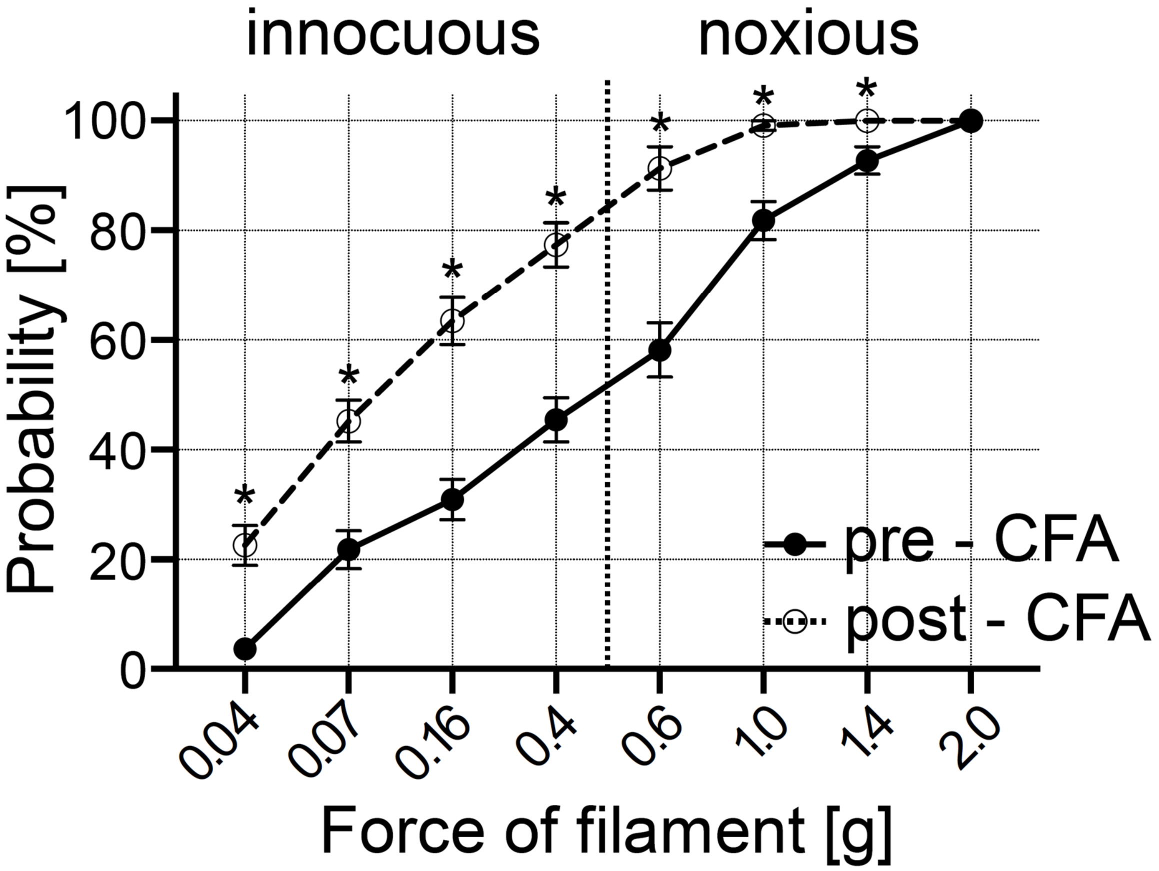

Supplementary Fig. 5: Complete Freund's adjuvant (CFA)-Inflammatory pain model. Related to Figures 2, 5 and 6.

Within animal comparison of paw withdrawal probabilities in response to graded von Frey stimulation of the hindpaw at Baseline pre-CFA (black line) and after injection of Complete Freund's adjuvant (Baseline post-CFA; dashed line) in the left hindpaw, which led to mechanical hypersensitivity to innocuous and noxious stimuli.

n = 22 mice; $p < 0.001$; two-way repeated measures ANOVA with post-hoc Bonferroni test. Exact F and p values in Supplementary Table 1. Data are shown as mean ± S.E.M. Source data for are provided as a Source Data file.

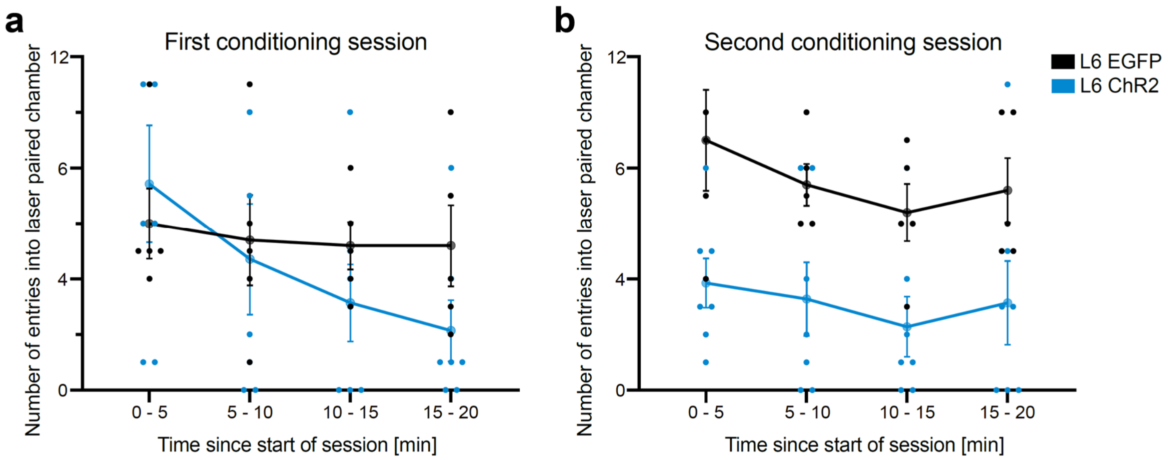

Supplementary Fig. 6. Reentry analysis of conditioned place aversion test (CPA) shows that stimulation of layer 6 corticothalamic neurons leads to both acute and learned aversion. Related to Figure 2.

Analysis of the number of entries to the laser-paired chamber within and across the two conditioning sessions (panels **(a)** and **(b)**, respectively), each session divided into four time intervals to track within-session changes. Data shows that for L6-ChR2 mice (1.) entries dropped throughout the first conditioning session and (2.) that already at the beginning of the second conditioning session, experimental L6-ChR2 mice started out at low entry levels, strongly indicating learned aversion.

L6-EGFP *n* = 5 and L6-ChR2 *n* = 7 mice. Data are shown as mean ± S.E.M. Source data for a, b are provided as a Source Data file.

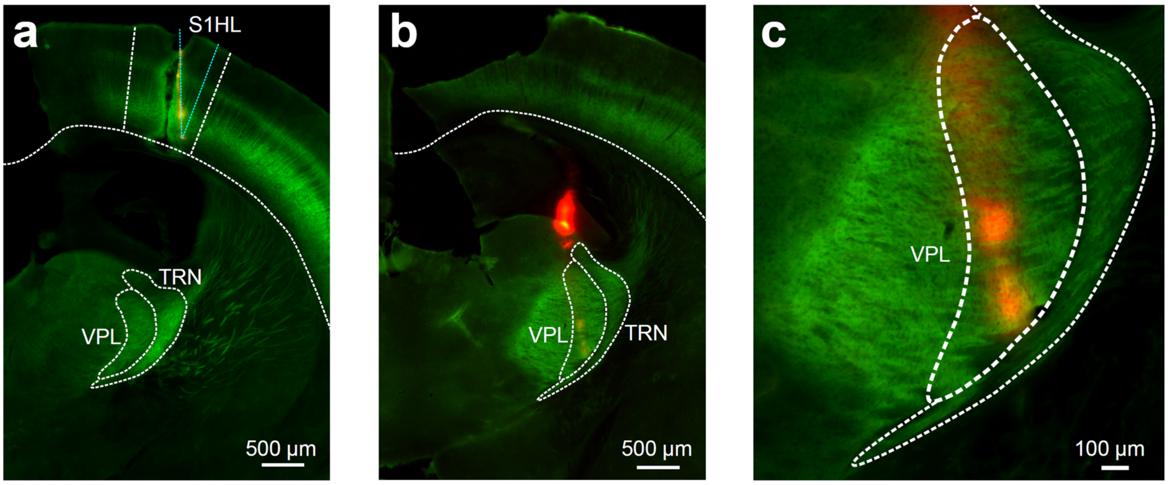

Supplementary Fig. 7. Recovery of silicon probe recording sites in S1 hindlimb cortex (S1HL) and ventral posterolateral thalamus (VPL). Related to Figures 3 and 4.

Example coronal sections of dyed (red) silicon probe location in **(a)** S1HL cortex and **(b, c)** VPL of Ntsr1-Cre-ChR2-EYFP (green) mouse. Representative example of 4 independent experiments with similar results.

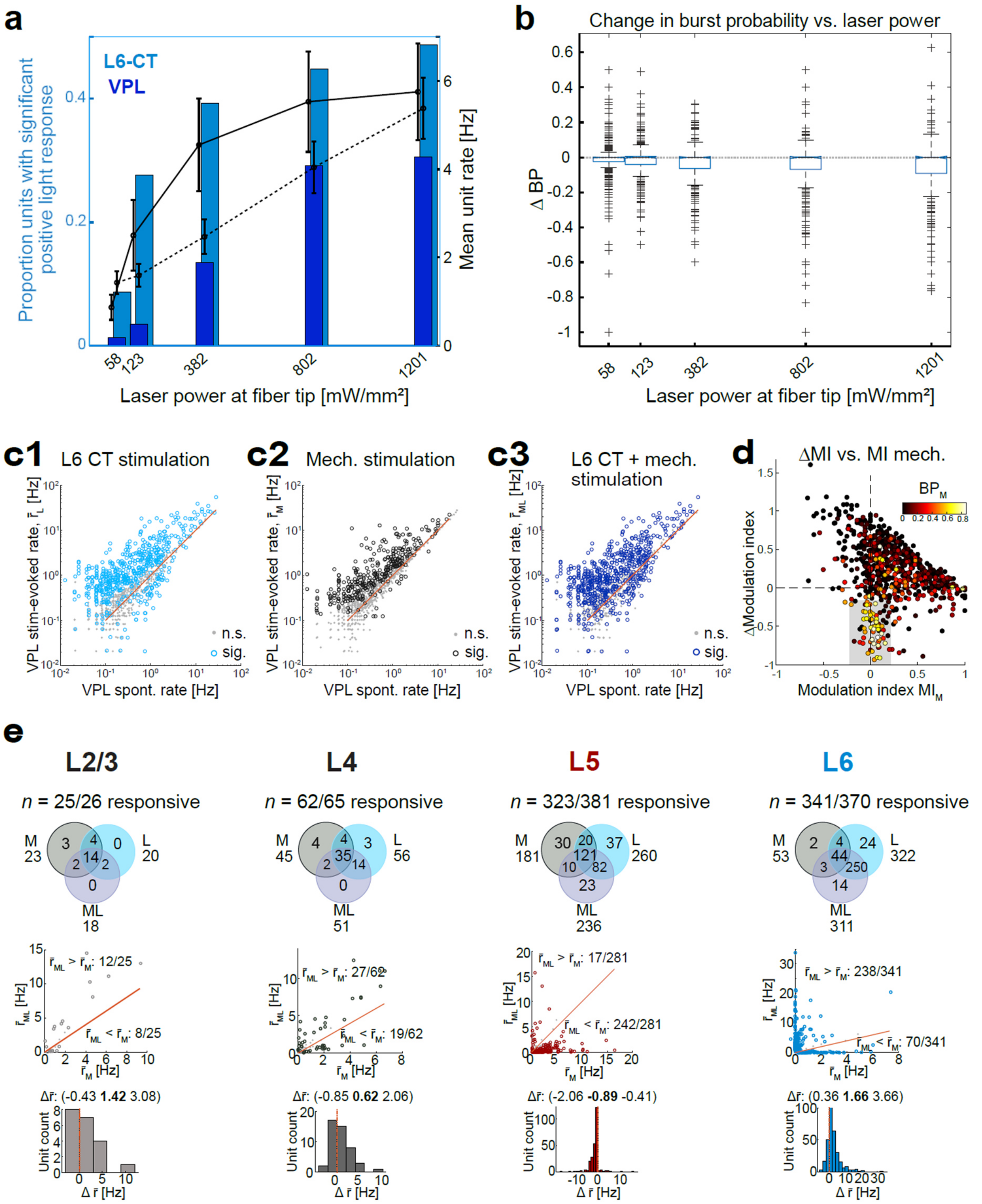

Supplementary Fig. 8. Layer 6 corticothalamic (L6-CT) stimulation (ChR2) effects on ventral posterolateral thalamus (VPL) and S1 hindlimb cortex (S1HL) activity. Related to Figures 3 and 4.

(a) Proportion of laser-responsive units (bar plots) and evoked firing rates (line plots) in L6-CT (blue bars, black line, *n* = 92 units) and VPL (dark blue bars, black dashed line, *n* = 169

units) recorded simultaneously as a function of laser power from an exemplar experiment (*n* = 4 mice). Data are shown as mean ± S.E.M.

(b) Change in bursting probability (ΔBP) (laser-evoked vs. baseline) in VPL is a function of laser power (*n* = 169 units), Kruskal-Wallis test, *p* = 0.013.

(c1,2,3) Stimulus-evoked spiking rate ($\bar{r}_L$, $\bar{r}_M$, $\bar{r}_{ML}$) vs. spontaneous spiking rate by VPL unit. Open markers: significantly modulated units in each condition (*n* = 536, 333, 623, for L, M, ML, respectively). Filled circles: not significantly modulated. Diagonal lines indicate equal responses. Central tendency and dispersion presented as (1st quartile **median** 3rd quartile): $\bar{r}_L$ (0.54 **1.07** 2.60) Hz, $\bar{r}_M$ (0.47 **0.99** 2.3) Hz, $\bar{r}_{ML}$ (0.69 **1.31** 3.24) Hz.

(d) Scatter plot: ΔMI vs. MI_M for individual VPL units ($\Delta MI = MI_{ML} - MI_M$). Each unit is colored by BP_M. A subset of bursty units had near-zero MI_M and $MI_{ML} < 0$ (gray box), suggesting that non-sensory coding VPL units are suppressed by L6-CT activation which further increases the overall proportion of sensory-driven VPL spike output.

(e) Breakdown of S1HL layer-specific population responses to L/M/ML conditions; **Upper row:** Population overlap of L/M/ML - encoding units in each layer. **Middle row:** comparison of $\bar{r}_{ML}$ vs. $\bar{r}_M$ for all units showing significant difference in firing rate between M and ML conditions (*p* < 0.05, signed rank). **Lower row:** distribution of $\Delta\bar{r} = \bar{r}_{ML} - \bar{r}_M$ for each unit, negative values correspond to L6-CT-suppressed units and positive values to L6-CT enhanced units. Layer 5 (L5) was significantly more suppressed than other layers (Supplementary Table 2). Source data for a-e are provided as a Source Data file.

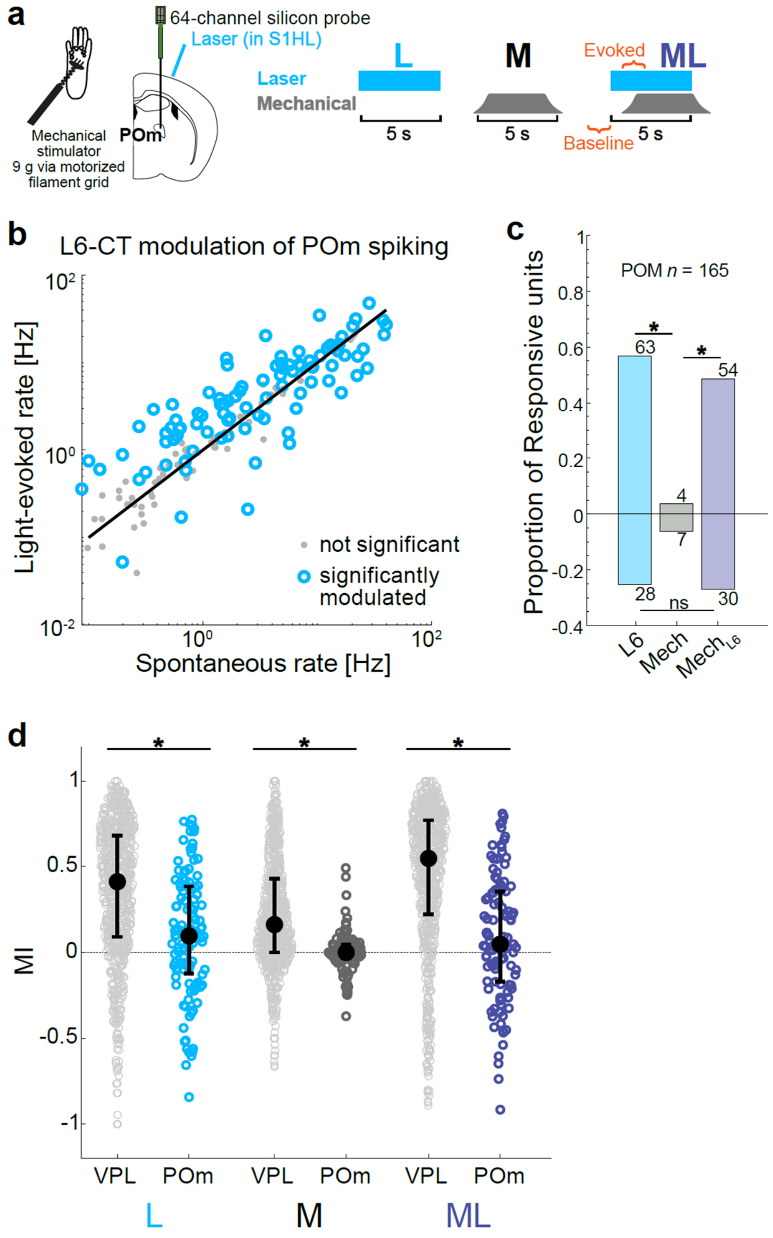

Supplementary Fig. 9. Heterogeneous effects of L6-CT activation on posterior medial (POm) thalamic units.

(a) Combined mechanical and L6-CT stimulation paradigm was identical to that used for VPL experiments (Fig. 3). 165 POm units were pooled from silicon probe recording in two anesthetized ChR2-expressing Ntsr1-Cre mice.

(b) Optogenetic stimulation of L6-CT neurons modulates spontaneous spiking in POm.

(c) Enhanced/suppressed fractions by condition. 111/165 neurons were modulated in at least one condition. 91/111 units were significantly modulated by light alone, with 63/91 enhanced and 28/91 suppressed. Two-sided X^2 test followed by Marascuillo procedure ($p < 0.01$). POm proportions significantly favored suppression (two-sided McNemar's test, $p < 0.01$) in comparison to VPL in all conditions (see VPL proportions in Fig. 3c).

(d) Comparison of stimulus-evoked modulation of VPL and POm units. L6-CT optogenetic stimulation alone and in combination with mechanical stimulus largely enhanced VPL spiking but had more mixed effects on POm spiking; POm (*n* = 111 units) was largely insensitive to mechanical stimulus alone compared to VPL (*n* = 742 units). VPL MI_L (0.09 **0.41** 0.68) > POm MI_L (-0.12 **0.10** 0.38); VPL MI_M (0.0 **0.16** 0.43) > POm MI_M (-0.03 **0.00** 0.05); VPL MI_{ML} (0.22 **0.55** 0.77) > POm MI_{ML} (-0.17 **0.05** 0.35) ($p < 0.001$).
Data shown as median and interquartile range. One-sided rank-sum test (right-tailed). * represents $p < 0.05$; exact *p* values in Supplementary Table 1. Source data for b-d are provided as a Source Data file.

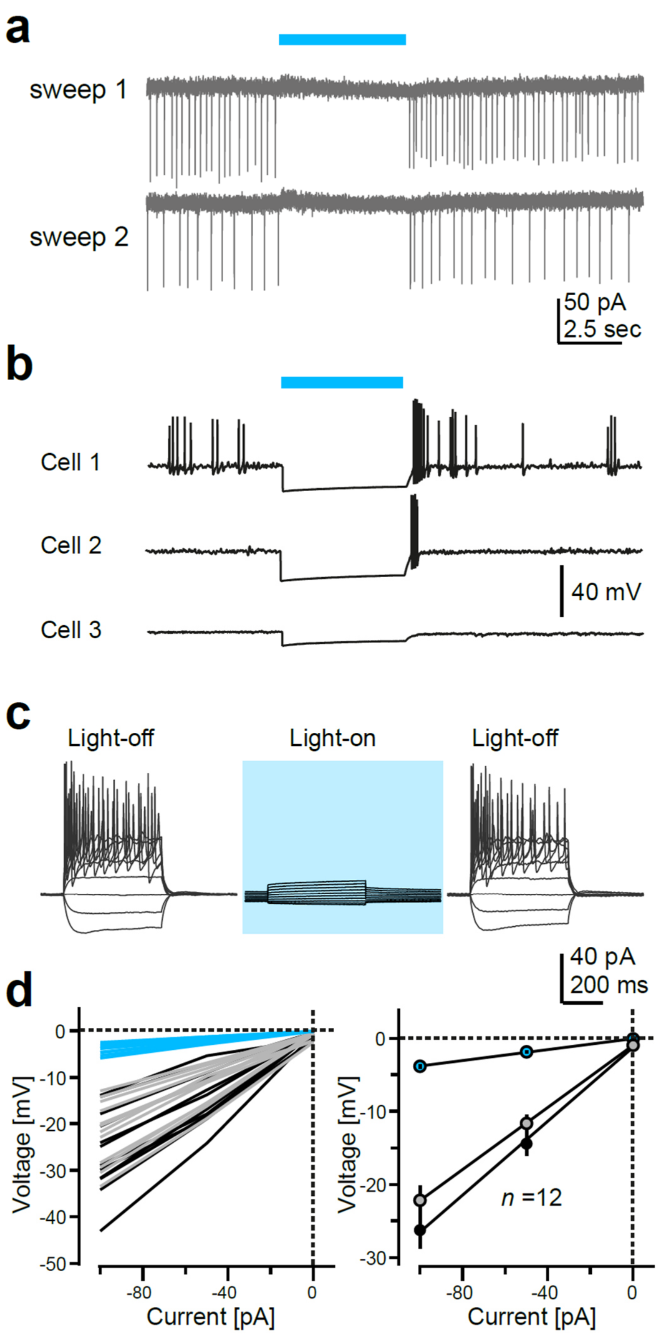

Supplementary Fig. 10. Validation of inhibitory opsin in L6 corticothalamic (L6-CT) S1 hindlimb cortex (S1HL) neurons: stGtACR2-expressing L6-CT neurons are strongly hyperpolarized in response to laser stimulation and spiking is efficiently suppressed.

(a) Effect of stGtACR2 activation on spontaneous spikes recorded in loose-cell attached mode.

(b) Effect of stGtACR2 activation on membrane potential, spontaneous action potential firing, and rebound potentials and spikes, recorded in current-clamp configuration. These exemplary cells were kept at ~40 mV by direct current injection through the recording pipette.

(c) A representative cell showing voltage responses triggered by injections of square pulses of current (500 ms, from -100 to +300 pA, 20 pA steps) before (left), during (middle), and after (right) stimulation with blue light.
(d) Left: Amplitude of membrane potential changes before (black), during (blue), and after (gray) light stimulation in stGtACR2-expressing L6-CT neurons (*n* = 12). Right: summary plot of the effect of light activation on the slope of the current-voltage relationship (input resistance) in all 12 neurons.
Data represented as means ± SEM. Source data for a-d are provided as a Source Data file.

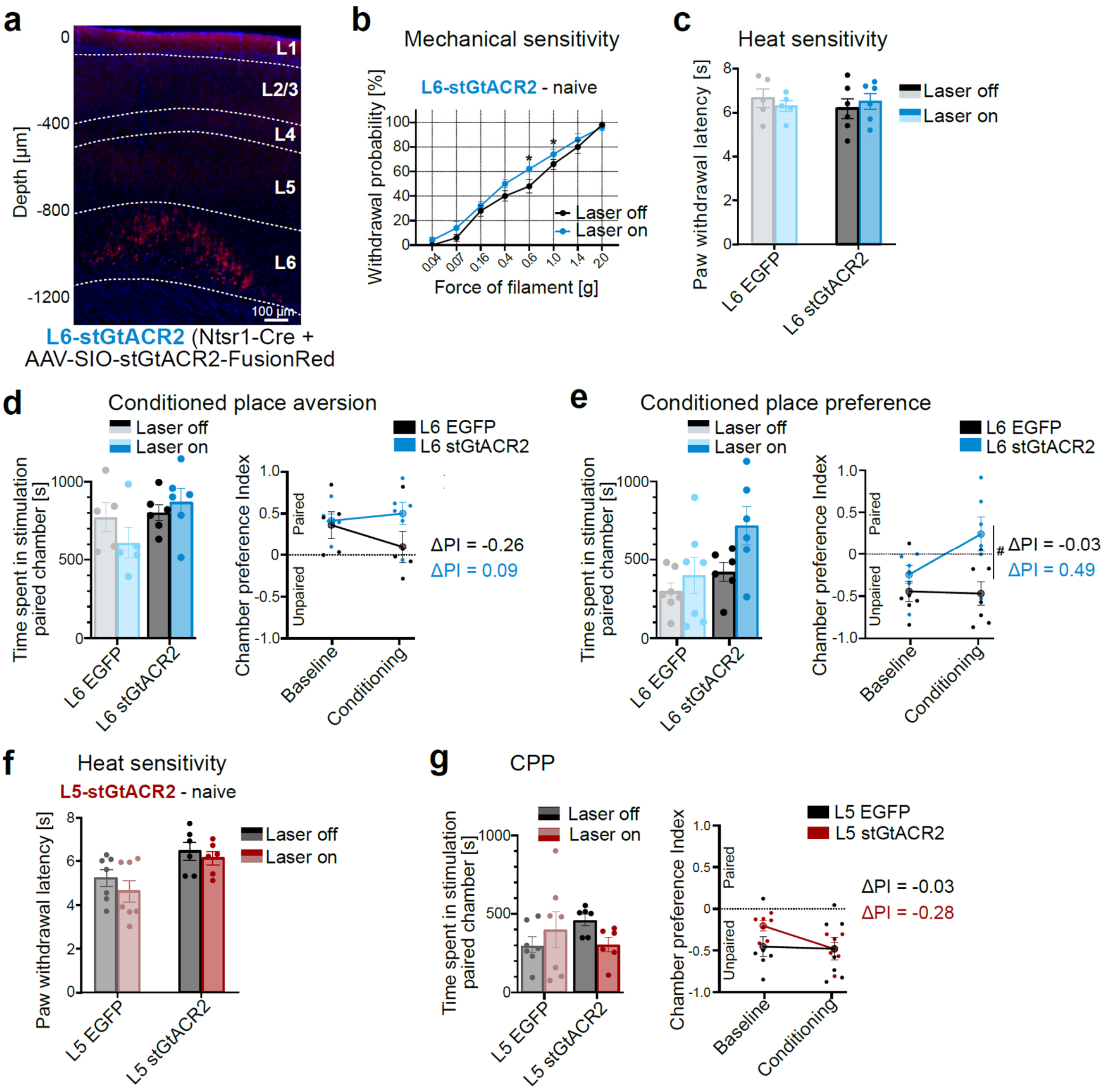

Supplementary Fig. 11. Optogenetic inhibition of L6 corticothalamic (L6-CT) and Layer 5 (L5) activity in the S1 hindlimb cortex (S1HL).

(a) Expression of stGtACR2-FusionRed (red) in L6 S1HL of a Ntsr1-Cre mouse showing fluorescence in L6-CT neurons. Depth is registered relative to S1HL layer borders (dashed lines, estimated based on soma sizes and densities using DAPI signals, blue). Representative example of *n* = 16 independent experiments with similar results.

(b) Within-animal comparison of paw withdrawal probabilities in response to graded von Frey stimulation of the hindpaw at baseline (black, laser off) and during optogenetic inhibition (blue, laser on, 5 s continuous pulse) in the contralateral S1HL of L6-stGtACR2 mice (*n* = 10).

(c) Paw withdrawal latencies in response to heat stimulation of the left hindpaw with (Laser on, blue, 5 s continuous pulse) and without (black, Laser off) optogenetic inhibition of L6-CT in the contralateral S1HL of L6-EGFP (*n* = 5) and L6-stGtACR2 (*n* = 6) mice.

(d) Conditioned place aversion (CPA). Population analysis of total time spent in the laser-paired chamber at baseline (Laser off, black/gray) and during inhibition (Laser on, blue 20 Hz laser stimulation in S1HL cortex) of L6-EGFP (n = 5) and L6-stGtACR2 (n = 6) naive mice. Average chamber preference indices (PI) for L6-stGtACR2 (n = 6) and L6-EGFP (n = 5) mice. A PI of 1 indicates a full preference for the paired chamber, while a PI of -1 indicates a full preference for the unpaired chamber, i.e. full avoidance of the laser-paired chamber. PIs were not significantly different between groups during laser stimulation or at baseline.
(e) Conditioned place preference (CPP). Population analysis of total time spent in the laser-paired chamber at baseline (Laser off, black/gray) and during inhibition (Laser on, blue 20 Hz laser stimulation in S1HL cortex) of L6-EGFP (n = 7) and L6-stGtACR2 (n = 6) mice with CFA-induced paw inflammation. Animals were injected with Complete Freund's adjuvant (CFA) (see Methods) one day before initiating the first baseline session. PIs for L6-stGtACR2 (n = 6) and L6-EGFP (n = 7) mice were significantly different between groups during laser stimulation (p = 0.0045), but not at baseline.
(f) Optogenetic inhibition of L5 activity in the S1HL cortex. Paw withdrawal latencies in response to heat stimulation of the left hindpaw with (Laser on, red, 5 s continuous pulse) and without (black, Laser off) optogenetic inhibition of L5 in the contralateral S1HL of L5-EGFP (n = 7) and L5-stGtACR2 (n = 6) mice.
(g) CPP. Population analysis of total time spent in the laser-paired chamber at baseline (Laser off, black/gray) and during inhibition (Laser on, red 20 Hz laser stimulation in S1HL cortex) of L5-EGFP (n = 7) and L5-stGtACR2 (n = 6) mice with CFA-induced paw inflammation. Animals were injected with CFA (see Methods) one day before initiating the first baseline session. PIs for L5-stGtACR2 (n = 6) and L5-EGFP (n = 7) were not significantly different between groups during laser stimulation or at baseline.

* and # represent $p < 0.05$; Supplementary Fig. 11 b-g: Two-way repeated measures ANOVA with post-hoc Bonferroni test. Exact F and p values in Supplementary Table 1. Data are shown as mean ± S.E.M. Source data for b-g are provided as a Source Data file.

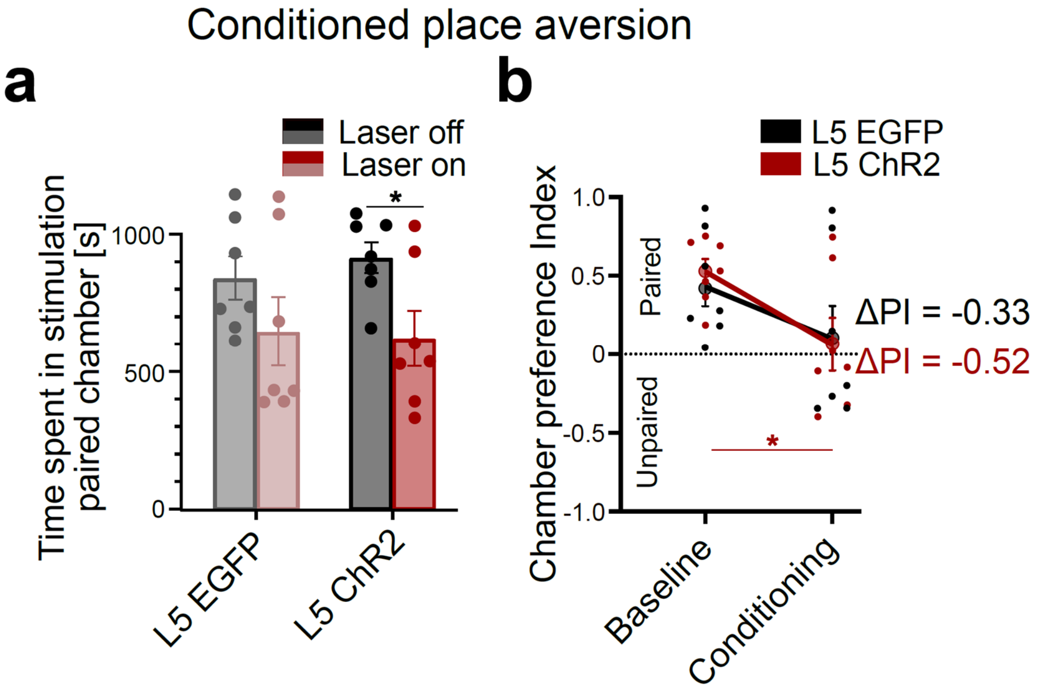

Supplementary Fig. 12. Layer 5 (L5) ChR2 conditioned place aversion (CPA) test. Related to Figure 6.

(a) Population analysis of total time spent in the laser-paired chamber at baseline (Laser off, black/gray) and during stimulation (Laser on, red 20 Hz laser stimulation in S1 hindlimb cortex (S1HL)) of L5-EGFP (n = 7) and L5-ChR2 (n = 7) mice.
(b) Average chamber preference indices (PI) for L5-ChR2 (n = 7) and L5-EGFP (n = 7) mice. A PI of 1 indicates a full preference for the paired chamber, while a PI of -1 indicates a full preference for the unpaired chamber, i.e. full avoidance of the laser-paired chamber. PIs were not significantly different between groups during laser stimulation or at baseline.

* represents $p < 0.05$; two-way repeated measures ANOVA with post-hoc Bonferroni test. Exact F and p values in Supplementary Table 1. Data are shown as mean ± S.E.M. Source data for a, b are provided as a Source Data file.

Repeating the real-time place aversion paradigm (CPA) from Fig. 2 but with L5 stimulation, shows that L5-ChR2 mice spent less time in the laser-paired chamber relative to the time spent in the same chamber during the baseline session (i.e. without optogenetic stimulation). However, the avoidance in L5-ChR2 animals was much less pronounced compared to L6-ChR2 animals (Fig. 2 h-j). Furthermore, the chamber preference index shows that this avoidance effect is indistinguishable between L5-ChR2 and L5-EGFP controls (Supplementary Fig. 9b) suggesting that the avoidance stems entirely from the laser light (as seen also in the L6-EGFP controls, Fig. 2j). We conclude that L5 activation is much less aversive, if at all, compared to L6-CT activation.

Figure	Group	*F*, *p* values, 95% confidence interval (*CI*)	Statistical test
2b	L6-ChR2 naive von Frey	*F* = 72.25; *p* = 1.36×10^{-5}<br>0.04 g; *p* = 0.357<br>0.07 g; *p* = 0.049<br>0.16 g; *p* = 0.035<br>0.4 g; *p* < 0.001<br>0.6 g; *p* = 0.001<br>1.0 g; *p* = 0.007<br>1.4 g; *p* = 0.567<br>2.0 g; *p* = 1<br>CI Baseline = 45.5 to 62<br>CI Laser = 63.3 to 78.2	Repeated measures ANOVA with post-hoc Bonferroni test
2c	L6-ChR2 CFA von Frey	*F* = 17.07; *p* = 0.002<br>0.04 g; *p* = 0.005<br>0.07 g; *p* = 0.02<br>0.16 g; *p* = 0.41<br>0.4 g; *p* = 1<br>0.6 g; *p* = 0.96<br>1.0 g; *p* = 1<br>1.4 g; *p* = 1<br>2.0 g; *p* = 1<br>CI Baseline = 71.7 to 84.3<br>CI Laser = 80.1 to 89.9	Repeated measures ANOVA with post-hoc Bonferroni test
2d	L6-ChR2 60% withdrawal thresholds	Baseline Naive - Laser Naive, *p* = 4.4×10^{-5}, CI -0.57 to -0.18<br>Baseline CFA - Laser CFA, *p* = 0.926, CI –0.24 to 0.15<br>Laser Naive - Laser CFA, *p* = 0.083, CI -0.02 to 0.38<br>Baseline Naive - Laser CFA, *p* = 2.54×10^{-8}, CI 0.36 to 0.76<br>Laser Naive - Baseline CFA, *p* = 0.268, CI -0.06 to 0.33<br>Baseline Naive - Baseline CFA, *p* = 1.59×10^{-7}, CI 0.32 to 0.71	Tukey's test
2g	L6-ChR2 CPA	*F* = 17.56, *p* = 0.002<br>L6-EGFP (control) *p* = 0.318, CI -121 to 452.1<br>L6-ChR2 (exper.) *p* = 0.002, CI 189.2 to 673.4	Two-way repeated measures ANOVA with post-hoc Bonferroni test
2h	L6-ChR2 CPA preference indices	*F* = 17.93, *p* = 0.002<br>Between timepoints (within groups):<br>L6-EYFP (control) *p* = 0.3946, CI -0.24 to 0.77<br>L6-ChR2 (exper.) *p* = 0.0012, CI 0.37 to 1.2<br>Between groups (within timepoints):<br>Baseline *p* = >0.999, CI -0.45 to 0.53<br>Conditioning *p* = 0.0227, CI 0.07 to 1.06	Two-way repeated measures ANOVA with post-hoc Bonferroni test
3c	VPL response fraction	X^2 test statistic = 1997.5<br>*p* = 2.2×10^{-16}	X^2 (two-sided) test followed by Marascuillo procedure
3e,f	Change in spiking rate per unit	*p* < 0.05<br>See Source Data for p-values for individual units.	Wilcoxon signed-rank or ZETA test

3g	VPL modulation index	$p = 2.38 \times 10^{-49}$<br>L-M: $p = 1.73 \times 10^{-15}$<br>L-ML: $p = 3.63 \times 10^{-10}$<br>M-ML: $p = 1.15 \times 10^{-43}$	Friedman test with post-hoc Wilcoxon signed-rank test
3h	VPL response probability	$p < 0.001$<br>L-M: $p = 0.002$<br>L-ML: $p < 0.001$<br>M-ML: $p < 0.001$	Friedman test with post-hoc Wilcoxon signed-rank test
3i	Change in BP per unit	$p < 0.05$<br>See Source Data for p-values for individual units.	McNemar's test
	BP_{ML} vs. BP_M	$p < 0.001$	Wilcoxon signed-rank test
4c	Cortex response fraction (L2/3)	X^2 test statistic = 2808.9<br>$p < 2.2 \times 10^{-16}$	X^2 (two-sided) test followed by Marascuillo procedure
	Cortex response fraction (L4)	X^2 test statistic = 2127.1<br>$p < 2.2 \times 10^{-16}$	X^2 (two-sided) test followed by Marascuillo procedure
	Cortex response fraction (L5)	X^2 test statistic = 628.4<br>$p < 2.2 \times 10^{-16}$	X^2 (two-sided) test followed by Marascuillo procedure
	Cortex response fraction (L6)	X^2 test statistic = 1261.3<br>$p < 2.2 \times 10^{-16}$	X^2 (two-sided) test followed by Marascuillo procedure
4e	Cortex modulation index (L2/3)	$p = 0.0019$<br>L-M; $p = 0.303$<br>L-ML; $p = 1.97 \times 10^{-5}$<br>M-ML; $p = 1$	Friedman test with post-hoc Wilcoxon signed-rank test
	Cortex modulation index (L4)	$p = 5.97 \times 10^{-5}$<br>L-M: $p = 0.221$<br>L-ML: $p = 3.27 \times 10^{-9}$<br>M-ML: $p = 1$	Friedman test with post-hoc Wilcoxon signed-rank test
	Cortex modulation index (L5)	$p = 6.48 \times 10^{-77}$<br>L-M: $p = 5.07 \times 10^{-49}$<br>L-ML: $p = 2.20 \times 10^{-12}$<br>M-ML: $p = 4.80 \times 10^{-47}$	Friedman test with post-hoc Wilcoxon signed-rank test

	Cortex modulation index (L6)	$p = 2.10 \times 10^{-12}$<br>L-M: $p = 7.44 \times 10^{-6}$<br>L-ML: $p = 0.51$<br>M-ML: $p = 4.59 \times 10^{-5}$	Friedman test with post-hoc Wilcoxon signed-rank test
	Change in spiking rate per unit	$p < 0.05$<br>See Source Data for p-values for individual units.	Wilcoxon signed-rank or ZETA test
4f	MIs across layers	L condition *p* values<br>**L4** **L5** **L6**<br>**L2/3** 0.1129 0.00030 0.00043<br>**L4** na 0.01418 0.00180<br>**L5** na na 0.00355<br><br>M condition *p* values<br>**L4** **L5** **L6**<br>**L2/3** 0.4521 2.1×10^{-6} 0.00030<br>**L4** na 6.3×10^{-10} 3.36×10^{-9}<br>**L5** na na 3.7×10^{-58}<br><br>ML condition *p* value<br>**L4** **L5** **L6**<br>**L2/3** 0.2828 4.24×10^{-11} 0.0232<br>**L4** na 1.97×10^{-23} 4.05×10^{-5}<br>**L5** na na 1.88×10^{-43}	Rank-sum test
5d	Median and 1st/3rd quartiles MI_L per layer	$p < 0.01$<br>L2/3: 14/29 (48%) units (MI_L -0.26 **-0.09** 0.05); L4: 25/52 (48%) units (MI_L 0.08 **0.22** 0.41); L5: 96/150 (65%) units (MI_L -0.69 **-0.23** 0.03); L6: 27/52 (52%) units (MI_L 0.07 **0.37** 0.52). L2/3 vs. L5 and L4 vs. L6. were not significant.	Rank-sum test
5e	L5-stGtACR2 von Frey	$F = 13.787$; $p = 0.014$<br>0.04 g; $p = 0.203$<br>0.07 g; $p = 0.004$<br>0.16 g; $p = 0.013$<br>0.4 g; $p = 0.041$<br>0. 6 g; $p = 0.102$<br>1.0 g; $p = 0.175$<br>1.4 g; $p = 0.465$<br>2.0 g; $p = 1$<br>CI Baseline = 33.3 to 54.2<br>CI Laser = 46.6 to 65.1	Two-way repeated measures ANOVA with post-hoc Bonferroni test
5f	L5-stGtACR2 CPA	$F = 11.75$, $p = 0.006$<br>L5-EYFP (control) $p = 0.316$, CI -138.7 to 528.1<br>L5-stGtACR2 (exper.) $p = 0.0150$, CI 93.9 to 814.1	Two-way repeated measures ANOVA with post-hoc Bonferroni test

5g	L5-stGtACR2 CPA preference indices	*F* = 11.69, *p* = 0.006<br>Between time points (within groups):<br>L5-EYFP (control) *p* = 0.3306, CI -0.25 to 0.91<br>L5-stGtACR2 (exper.) *p* = 0.0146, CI 0.17 to 1.42<br>Between groups (within time points):<br>Baseline *p* = >0.999, CI -0.81 to 0.59<br>Conditioning *p* = 0.4805, CI 0.35 to 1.05	Two-way repeated measures ANOVA with post-hoc Bonferroni test
6e	L5-ChR2 naive von Frey	*F* = 273.49; *p* = $1.48x10^{-5}$<br>0.04 g; *p* = 1<br>0.07 g; *p* = 0.007<br>0.16 g; *p* = 0.003<br>0.4 g; *p* = 0.003<br>0.6 g; *p* = 0.0003<br>1.0 g; *p* = 0.003<br>1.4 g; *p* = 0.003<br>2.0 g; *p* = 0.012<br>CI Baseline = 44.7 to 64.9<br>CI Laser = 12.8 to 26.8	Two-way repeated measures ANOVA with post-hoc Bonferroni test
6f	L5-ChR2 CFA von Frey	*F* = 19.317; *p* = 0.001<br>0.04 g; *p* = 0.189<br>0.07 g; *p* = 0.007<br>0.16 g; *p* = 0.028<br>0.4 g; *p* = 0.035<br>0.6 g; *p* = 0.084<br>1.0 g; *p* = 0.105<br>1.4 g; *p* = 0.777<br>2.0 g; *p* = 1<br>CI Baseline = 65.1 to 78.6<br>CI Laser = 46.7 to 62.5	Two-way repeated measures ANOVA with post-hoc Bonferroni test
6g	L5-ChR2 60% withdrawal thresholds	Baseline Naive - Laser Naive, *p* = 0.0004, CI 0.46 to 1.82<br>Baseline CFA - Laser CFA, *p* = 0.03, CI 0.038 to 0.996<br>Laser Naive - Laser CFA, *p* = 0.0001, CI -0.49 to 1.66<br>Baseline Naive - Laser CFA, *p* = 0.99, CI -0.52 to 0.65<br>Laser Naive - Baseline CFA, *p* = $1.33x10^{-7}$, CI 1.01 to 2.18<br>Baseline Naive - Baseline CFA, *p* = 0.177, CI -0.13 to 1.04	Tukey's test
6i	L5-ChR2 CPP	*F* = 7.7572, *p* = 0.022<br>L5-EGFP (control) *p* = >0.999, CI -225.6 to 147.4<br>L5-ChR2 (exper.) *p* = 0.0213, CI -448.7 to 40.09	Two-way repeated measures ANOVA with post-hoc Bonferroni test
6j	L5-ChR2 CPP preference indices	*F* = 5.334, *p* = 0.046<br>Between timepoints (within groups):<br>L6-EYFP (control) *p* = >0.999<br>L6-ChR2 (exper.) *p* = 0.0298<br>Between groups (within timepoints):<br>Baseline *p* = 0.6967, CI -0.46 to 0.20<br>Conditioning *p* = 0.0019, CI -0.86 to -0.20	Two-way repeated measures ANOVA with post-hoc Bonferroni test

7a	L5 vs L6-CT withdrawal probability (% change from baseline)	*F* = 114.6; *p* = $3.99x10^{-8}$<br>0.04 g; *p* = 0.4080<br>0.07 g; *p* = 0.0013<br>0.16 g; *p* = 0.0016<br>0.4 g; *p* = 0.0055<br>0.6 g; *p* = 0.0014<br>1.0 g; *p* = 0.0090<br>1.4 g; *p* = 0.3384<br>2.0 g; *p* = 0.1437<br>CI = -52.92 to -35.25	Two-way repeated measures ANOVA with post-hoc Bonferroni test
7b	AUC L5 and L6-CT, Naive and CFA	Naive:<br>*F* = 57.85; *p* = $2x10^{-6}$<br>*p* L5 = $1.77x10^{-9}$<br>*p* L6 = $5.04x10^{-5}$<br>CI L5 = 70.01 to 99.6<br>CI L6 = -39.55 to -16.63<br><br>CFA:<br>*F* = 7.69; *p* = 0.012<br>L5 *p* = 0.0001<br>L6 *p* = 0.841<br>L5 CI = 15.78 to 45.30<br>L6 CI = -21.66 to 10.68	Two-way repeated measures ANOVA with post-hoc Bonferroni test
S3b	L6-ChR2 Hargreaves	*F* = 9.51; *p* = 0.012<br>L6-EGFP (control) *p* = 0.92, CI -0.87 to 1.59<br>L6-ChR2 (exper.) *p* = 0.006, CI 0.49 to 2.56	Two-way repeated measures ANOVA with post-hoc Bonferroni test
S3c	L5-ChR2 Hargreaves	*F* = 2.15; *p* = 0.169<br>L6-EGFP (control) *p* = 0.55, CI -0.74 to 1.94<br>L6-ChR2 (exper.) *p* = 0.75, CI -0.86 to 1.82	Two-way repeated measures ANOVA with post-hoc Bonferroni test
S4a	L5-EGFP control von Frey	*F* = 0.625; *p* = 0.465<br>0.04 g; *p* = 1<br>0.07 g; *p* = 1<br>0.16 g; *p* = 0.651<br>0.4 g; *p* = 1<br>0.6 g; *p* = 1<br>1.0 g; *p* = 1<br>1.4 g; *p* = 1<br>2.0 g; *p* = 1<br>CI Baseline = 38.6 to 58.9<br>CI Laser = 36.5 to 57.7	Two-way repeated measures ANOVA with post-hoc Bonferroni test
S4b	L6-EGFP control von Frey	*F* = 1.88; *p* = 0.229<br>0.04 g; *p* = 1<br>0.07 g; *p* = 1<br>0.16 g; *p* = 1<br>0.4 g; *p* = 1<br>0.6 g; *p* = 1<br>1.0 g; *p* = 1<br>1.4 g; *p* = 1<br>2.0 g; *p* = 1<br>CI Baseline = 34.3 to 55.7	Two-way repeated measures ANOVA with post-hoc Bonferroni test

		CI Laser = 42 to 63	
S4c	L5-EGFP control von Frey CFA	*F* = 0.04; *p* = 0.847<br>0.04 g; *p* = 1<br>0.07 g; *p* = 1<br>0.16 g; *p* = 1<br>0.4 g; *p* = 1<br>0.6 g; *p* = 1<br>1.0 g; *p* = 1<br>1.4 g; *p* = 1<br>2.0 g; *p* = 1<br>CI Baseline = 68.2 to 83.7<br>CI Laser = 68.8 to 84.3	Two-way repeated measures ANOVA with post-hoc Bonferroni test
S4d	L6-EGFP control von Frey CFA	*F* = 0.115; *p* = 0.791<br>0.04 g; *p* = 1<br>0.07 g; *p* = 1<br>0.16 g; *p* = 1<br>0.4 g; *p* = 1<br>0.6 g; *p* = 1<br>1.0 g; *p* = 1<br>1.4 g; *p* = 1<br>2.0 g; *p* = 1<br>CI Baseline = 57.6 to 77.4<br>CI Laser = 56.7 to 75.8	Two-way repeated measures ANOVA with post-hoc Bonferroni test
S5	L6-CT pre-/post-CFA	*F* = 57.43; *p* = 1.94×10^{-7}<br>0.04 g; *p* = 0.001<br>0.07 g; *p* = 0.003<br>0.16 g; *p* = 7.28×10^{-5}<br>0.4 g; *p* = 3.84×10^{-6}<br>0.6 g; *p* = 0.0004<br>1.0 g; *p* = 0.0006<br>1.4 g; *p* = 0.008<br>2.0 g; *p* =1<br>CI Pre-CFA = 48.9 to 59.7<br>CI Post-CFA = 70 to 79.3	Two-way repeated measures ANOVA with post-hoc Bonferroni test
S8b	VPL burst probability vs. laser strength	X^2 test statistic = 12.06<br>*p* = 0.0134<br>df =4	Kruskal-Wallis test.
S9b	Change in spiking rate per POm unit	*p* < 0.05<br>See Source Data for *p*-values for individual units.	Wilcoxon signed-rank or ZETA test
S9c	L6-CT enhanced/suppressed ratios for POm	X^2 test statistic = 301.7<br>*p* = 1.79×10^{-60}	X^2 (two-sided) test followed by Marascuillo procedure
	Suppression/ enhancement ratios between VPL and POm per condition	*p* = 4.0×10^{-7}<br>*p* = 0<br>*p* = 0.0011	Two-sided McNemar’s test

S9d	L6-CT MIs between VPL and POm per condition	*p* = 1.5 x 10^{-10}<br>*p* = 9.1 x 10^{-16}<br>*p* = 1.4 x 10^{-18}	One-sided rank-sum test
S11b	L6-stGtACR2 von Frey	*F* =15.059; *p* = 0.004<br>0.04 g; *p* = 0.168<br>0.07 g; *p* =0.104<br>0.16 g; *p* = 0.343<br>0.4 g; *p* 0.096<br>0. 6 g; *p* =0.01<br>1.0 g; *p* = 0.037<br>1.4 g; *p* = 0.081<br>2.0 g; *p* = 0.343<br>CI Baseline = 38.1 to 53.4<br>CI Laser = 44.8 to 59.7	Two-way repeated measures ANOVA with post-hoc Bonferroni test
S11c	L6-stGtACR2 Hargreaves	*F* = 0.022, *p* = 0.88<br>L5-EGFP (control) *p* = 0.252, CI -0.21 to 0.93<br>L5-ChR2 (exper.) *p* = 0.277, CI -0.84 to 0.21	Two-way repeated measures ANOVA with post-hoc Bonferroni test
S11d	L6-stGtACR2 CPA	*F* = 0.7378, *p* = 0.41<br>L6-EYFP (control) *p* = 0.159, CI -59.15 to 390.1<br>L6-stGtACR2 (exper.) *p* = 0.79, CI -273,2 to 136.9	Two-way repeated measures ANOVA with post-hoc Bonferroni test
	L6-stGtACR2 CPA preference indices	*F* = 0.8484, *p* = 0.381<br>Between timepoints (within groups):<br>L6-EYFP (control) *p* = 0.1936, CI -0.12 to 0.65<br>L6-stGtACR2 (exper.) *p* = >0.999, CI -0.43 to 0.26<br>Between groups (within timepoints):<br>Baseline *p* = >0.999, CI -0.54 to 0.43<br>Conditioning *p* = 0.1159, CI -0.89 to 0.08	Two-way repeated measures ANOVA with post-hoc Bonferroni test
S11e	L6-stGtACR2 CPP	*F* = 5.445, *p* = 0.0396<br>L6-EYFP (control) *p* = 0.8006, CI -402.8 to 199.6<br>L6-stGtACR2 (exper.) *p* = 0.0743, CI -622.7 to 27.99	Two-way repeated measures ANOVA with post-hoc Bonferroni test
	L6-stGtACR2 CPP preference indices	*F* = 2.332, *p* = 0.155<br>Between timepoints (within groups):<br>L6-EYFP (control) *p* = >0.999, CI -0.51 to 0.56<br>L6-stGtACR2 (exper.) *p* = 0.1011, CI -1.07 to 0.09<br>Between groups (within timepoints):<br>Baseline *p* = 0.6890, CI -0.696 to 0.297<br>Conditioning *p* = 0.0045, CI -1.21 to -0.22	Two-way repeated measures ANOVA with post-hoc Bonferroni test
S11f	L5-stGtACR2 Hargreaves	*F* = 5.798, *p* = 0.0347<br>L5-EGFP (control) *p* = 0.0812, CI -0.07 to 1.27<br>L5-ChR2 (exper.) *p* = 0.5622, CI -0.41 to 1.04	Two-way repeated measures ANOVA with post-hoc Bonferroni test

S11g	L5-stGtACR2 CPP	*F* = 0.3959, *p* = 0.54<br>L5-EYFP (control) *p* = 0.232, CI -256.1 to 52.87<br>L5-stGtACR2 (exper.) *p* = 0.066, CI -10.1 to 323.6	Two-way repeated measures ANOVA with post-hoc Bonferroni test
	L5-stGtACR2 CPP preference indices	*F* = 3.298, *p* = 0.09<br>Between timepoints (within groups):<br>L5-EYFP (control) *p* = >0.999<br>L6-stGtACR2 (exper.) *p* = 0.089<br>Between groups (within timepoints):<br>Baseline *p* = 0.236, CI -0.62 to 0.12<br>Conditioning *p* = >0.999, CI -0.37 to 0.38	Two-way repeated measures ANOVA with post-hoc Bonferroni test
S12a	L5 ChR2 CPA	*F* = 17.56, *p* = 0.01<br>L6-EGFP (control) *p* = 0.218, CI -93.1 to 482.5<br>L6-ChR2 (exper.) *p* = 0.045, CI 6.0 to 581.6	Two-way repeated measures ANOVA with post-hoc Bonferroni test
S12b	L5 ChR2 CPA preference indices	*F* = 9.677, *p* = 0.009<br>Between timepoints (within groups):<br>L5-EYFP (control) *p* = 0.2207, CI -0.16 to 0.83<br>L5-ChR2 (exper.) *p* = 0.0404, CI 0.02 to 1.01<br>Between groups (within timepoints):<br>Baseline *p* = 0.9960, CI -0.67 to 0.37<br>Conditioning *p* = >0.999, CI -0.48 to 0.55	Two-way repeated measures ANOVA with post-hoc Bonferroni test

Supplementary table 1. Description of statistical parameters and *p*- and *F*-values by figure.

	L	*M*	*ML*
L2/3 v L6	**127 (0.029)**	**-75 (0.0004)**	53 (1)
L4 v L6	**184 (8.5×10^{-6})**	**-69 (0.00252)**	74 (1)
L5 v L6	**190 (3.26×10^{-75})**	**-57 (1.14×10^{-5})**	**235 (1.32×10^{-64})**
L4 v L5	**-414 (1.37×10^{-10})**	-30 (1)	**-227 (3.11×10^{-22})**
L2/3 v L5	**-351 (5.57×10^{-6})**	-43 (0.164)	**-212 (1.51×10^{-11})**
L2/3 v L4	-20 (1)	-19 (0.893)	-13 (1)

Modulation Indices: Two-way mixed model ANOVA, *F* layer = 125.521, *p* layer <0.0001, *F* Condition = 41.752, *p* Condition <0.0001, *F* Interaction = 41.752, *p* Interaction <0.0001

	L	*M*	*ML*
L2/3 v L6	122 (1)	**-89 (0.0005)**	-7 (1)
L4 v L6	145 (0.0981)	**-88 (1.34×10^{-5})**	56 (1)
L5 v L6	**6610 (2.46×10^{-54})**	**-84 (1.22×10^{-5})**	**1345 (4.71×10^{-57})**
L4 v L5	-96 (0.398)	-23 (1)	**-89 (0.0347)**
L2/3 v L5	-97 (0.0829)	-33 (1)	**-94 (0.00496)**
L2/3 v L4	-9 (1)	-13 (1)	-41 (1)

Evoked firing rate $\bar{r}$: Two-way mixed model ANOVA, *F* layer = 10.097, *p* layer < 0.0001, *F* Condition = 10.473, *p* Condition = 0.001, *F* Interaction = 40.116, *p* Interaction < 0.0001

Supplementary table 2. Comparison of modulation index (top) and evoked firing rates (bottom) across cortical layers for L, M, and ML conditions (L6-CT activation). Values shown are percentage change of median MI or $\bar{r}$ values calculated as [(V1 - V2)/V2]. Significant values are shown in bold, relative enhancement in green, and relative suppression in red. For example, L4 vs. L5 ML = -89 indicates that L5 was suppressed relative to L4 for the ML condition.

Optogenetic manipulation	Test	Effect	Number of mice	*p* value
L6-ChR2	Von Frey	increased sensitivity	n = 10	$p = 1.36 \times 10^{-5}$
	Hargreaves	increased sensitivity	n = 7	p = 0.021
	CPA	aversion	n = 7	p = 0.0298
L5-ChR2	Von Frey	decreased sensitivity	n = 6	$p = 1.48 \times 10^{-5}$
	Hargreaves	no effect	n = 6	p = 0.231
	CPA	no effect (only laser aversion)	n = 7	p = 0.312
	CPP	place preference	n = 5	p = 0.045
L6-stGtACR2	Von Frey	small increase in sensitivity	n = 10	p = 0.004
	Hargreaves	no effect	n = 6	p = 0.88
	CPA	no effect	n = 6	p = 0.7904
	CPP	no effect	n = 6	p = 0.074
L5-stGtACR2	Von Frey	increased sensitivity	n = 6	p = 0.014
	Hargreaves	no effect	n = 6	p = 0.5622
	CPA	aversion	n = 6	p = 0.0150
	CPP	no effect	n = 6	p = 0.0662

Supplementary table 3. Summary of the effects of optogenetic manipulations of L6-CT and L5 activity on mechanical and thermal sensitivity, conditioned place preference and conditioned place aversion. Two-way ANOVA for repeated measures with Bonferroni tests for multiple comparisons were used.

Supplementary References

1. Schmitt, L. I. *et al.* Thalamic amplification of cortical connectivity sustains attentional control. *Nature* **545**, 219–223 (2017).
2. Cheah, M., Fawcett, J. W. & Andrews, M. R. Assessment of Thermal Pain Sensation in Rats and Mice Using the Hargreaves Test. *Bio Protoc* **7**, (2017).
