# Supplementary material for: Primary somatosensory cortex bidirectionally modulates sensory gain and nociceptive behavior in a layer-specific manner
Source: Nat Commun. 2023 May 24;14:2999. doi: 10.1038/s41467-023-38798-7 (PMC10209111; doi:10.1038/s41467-023-38798-7)
Supplement: Supplementary file 3 — Description of Additional Supplementary Files [file 41467_2023_38798_MOESM3_ESM.pdf]

Description of Additional Supplementary Files

File Name: Supplementary Movie 1
Description: **Optogenetically-evoked L6-CT activity in the S1HL cortex elicits spontaneous nocifensive behavior. Related to Fig. 1e, f, 6d.** Optogenetic stimulation of L6-CT neurons in S1HL of a L6-ChR2 mouse with fiber optic implant elicits lifting and shaking of left hindpaw.

File Name: Supplementary Movie 2
Description: **Optogenetically-evoked L5 activity in the S1HL cortex does not elicit nocifensive behavior. Related to Fig. 6d.** Optogenetic stimulation of L5 neurons in S1HL of a L5-ChR2 mouse with fiber optic implant does not elicit obvious behavioral reactions.
