# Supplementary material for: Primary somatosensory cortex bidirectionally modulates sensory gain and nociceptive behavior in a layer-specific manner
Source: Nat Commun. 2023 May 24;14:2999. doi: 10.1038/s41467-023-38798-7 (PMC10209111; doi:10.1038/s41467-023-38798-7)
Supplement: Supplementary file 6 — Reporting Summary [file 41467_2023_38798_MOESM6_ESM.pdf]

nature portfolio

Corresponding author(s): Alexander Groh

Last updated by author(s): Mar 9, 2023

Reporting Summary

Nature Portfolio wishes to improve the reproducibility of the work that we publish. This form provides structure for consistency and transparency in reporting. For further information on Nature Portfolio policies, see our Editorial Policies and the Editorial Policy Checklist.

Statistics

For all statistical analyses, confirm that the following items are present in the figure legend, table legend, main text, or Methods section.

n/a	Confirmed	
☐	☒	The exact sample size (*n*) for each experimental group/condition, given as a discrete number and unit of measurement
☐	☒	A statement on whether measurements were taken from distinct samples or whether the same sample was measured repeatedly
☐	☒	The statistical test(s) used AND whether they are one- or two-sided<br>*Only common tests should be described solely by name; describe more complex techniques in the Methods section.*
☒	☐	A description of all covariates tested
☐	☒	A description of any assumptions or corrections, such as tests of normality and adjustment for multiple comparisons
☐	☒	A full description of the statistical parameters including central tendency (e.g. means) or other basic estimates (e.g. regression coefficient) AND variation (e.g. standard deviation) or associated estimates of uncertainty (e.g. confidence intervals)
☐	☒	For null hypothesis testing, the test statistic (e.g. *F*, *t*, *r*) with confidence intervals, effect sizes, degrees of freedom and *P* value noted<br>*Give P values as exact values whenever suitable.*
☒	☐	For Bayesian analysis, information on the choice of priors and Markov chain Monte Carlo settings
☒	☐	For hierarchical and complex designs, identification of the appropriate level for tests and full reporting of outcomes
☒	☐	Estimates of effect sizes (e.g. Cohen's *d*, Pearson's *r*), indicating how they were calculated

Our web collection on statistics for biologists contains articles on many of the points above.

Software and code

Policy information about availability of computer code

Data collection

Behavioral experiments were conducted during the light cycle and experimenters were blinded to the experimental identity of the animals. Behavioral readouts were measured with von Frey test (Aesthesio Precise Tactile Sensory Evaluator, Ugo Basile S.R.L.), Thermal test (Ugo Basile Inc., Italy) and conditioned place aversion test (AnyMaze software, Version 7.1, Stoelting Co., Ireland).

Optogenetic stimulation (Behavior): The implanted fiber was coupled to an optical patch cord (Thorlabs GmbH) attached to a laser output module (473 nm) (Shanghai Laser Optics Century Co., Ltd.). The laser power at the fiber tip was measured with a power energy meter (Thorlabs GmbH). Irradiance values for layers 5 and 6 were estimated based on previous measurements in mammalian brain tissue 70. For 10 mW measured at the fiber tip (fiber NA = 0.39; fiber radius = 100 µm), the irradiance is 318.18 mW/mm², which corresponds to 3.47 mW/mm² at the level of L5 (0.75 mm cortical depth) and 1.54 mW/mm² at the level of L6 (1 mm cortical depth).

In vitro electrophysiology and optogentic stimulation: Slices were placed on a RC-27 chamber (Sutter Instruments) mounted under BX51 upright microscope (Olympus), equipped with DIC and fluorescent capabilities. Slices were maintained at 24±1 ºC using a dual TC344B temperature control system (Sutter Instruments). S1HL slices were continuously perfused with oxygenated (95%O2/ 5%CO2) ASCF solution containing (in mM): 125 NaCl, 2.5 KCl, 0.1 MgCl2, 4 CaCl2, 25 glucose, 1.25 NaH2PO4, 0.4 ascorbic acid, 3 myo-inositol, 2 Na-pyruvate, 25 NaHCO3, pH 7.4., and 315 mOsm. Cells were approached and patched under DIC, using 3.0±0.5 MegaOhm glass pipettes (WPI, Inc), pulled with a PC10 puller (Narishige, Japan). Recording pipettes were filled with a current clamp internal solution containing (in mM): 125 K-gluconate, 20 KCl, 10 HEPES, 0.5 EGTA, 4 ATP-Magnesium, 0.3 GTP-Sodium, 10 Na-Phosphocreatine, osmolarity: 312 mOsmol; pH 7.2 adjusted with KOH. In all recordings, we used a Multiclamp 700B amplifier (Axon instruments, Inc) controlled by Clampex 10.1 and Digidata 1440 digitizer (Molecular Devices, Inc). Detection and analysis of current-clamp recordings was done with Clampfit 10.1.
To assess the impact of stGtACR2 activation on L6-CT neuron activity, cells expressing red fluorescence were approached and recorded in either loose cell-attached or in whole-cell current clamp configuration. Cells displaying spontaneous spikes in cell-attached mode, were

challenged with pulses of blue light (480 nm, 5 sec), generated via a CoolLED illumination system (pE-300) controlled by a TTL pulse. In whole cell-current clamp mode, cells were maintained at near resting potentials (~-70 mV) or at more depolarized potentials (~-40 mV) via direct current injection through the patch pipette. Two experiments were performed in current clamp mode. First, to assess the impact of stGtACR2 activation on membrane potential and spontaneous spiking activity, cells were challenged with long (5 sec) pulses of blue light (480 nm), similar to the cell-attached experiments described above. Second, to determine if stGtACR2 activation leads to changes in the input resistance of L6 neurons, cells maintained at near resting potentials were stimulated with square pulses of current (500 ms, from -100 to +300 pA, 20 pA steps), and the amplitude of membrane potential changes before, during, and after blue light activation, was determined.

Electrophysiological recordings in vivo were done in anesthetized mice with sharpened 64-channel silicon probes (impedance ~50 kOhm) (Cambridge Neurotech), recorded with RHD2164 headstage amplifier chip (Intan technologies). Signals were amplified and digitized at a sampling rate of 30,030 Hz via an RDH2000 Intan evaluation board using USB 2.0 interface. An Intan Talker module (Cambridge Electronic Devices, Cambridge, UK) was for data acquisition with Spike 2 (Version 9.06) software. Mechanical stimulation was automated by a stimulation protocol prepared in Spike2 through an interface hardware (Power1401, Cambridge Electronic Design, Cambridge, UK) through a stepper controller, which initiated a motor (Mercury Step C-663 Stepper Motor Controller, PIMikroMove, Version 2.25.2.0).

Optogenetic stimulation (in vivo Electrophysiology): An optical fiber (Thorlabs GmbH, NA = 0.22; radius = 52.5 µm) was positioned ~0.5 mm perpendicular above the craniotomy. Laser power densities overlapped with the behavioral experiments (~0.5 - 27 mW at fiber tip corresponding to 57.72 - 3105.34 mW/mm² and 0.26 - 14.16 mW/mm² at the level of L6 70). Light pulses were initiated automatically by a stimulation protocol prepared in Spike2 through an interface hardware (Power1401, Cambridge Electronic Design, Cambridge, UK). Trial repetitions per condition were 31-52 trials for VPL recordings and 32-35 trials for S1 recordings.

Code and data necessary to reproduce the Matlab-generated figures in this study are provided at https://doi.org/10.11588/data/D2O0JZ and maintained at https://github.com/GrohLab/Ziegler_et_al_2023.

Data analysis

Statistics and Data Analysis
All behavioral data are expressed as the mean ± the standard error, and were analyzed using SPSS (Version 28.0.1.0) and R Studio (Version 4.2.0). Unless stated otherwise, two-way ANOVA for repeated measures with Bonferroni tests for multiple comparisons were used. A p-value less than 0.05 was considered to be significant. Microscopy images were edited using Fiji/Image J (Version 1.53c). Schematics and figures were created in Affinity Designer (Version 1.10.6), GraphPad Prism (Version 9.1.1), and Matlab 2022a.

Spike Sorting
Voltage data were band-pass filtered (500-5000 Hz). Spike2 data files (.smrx) were converted into binary files. The file conversion consisted in reading the electrophysiology channels in the .smrx file, transforming them back into uint16 values from the 16-bit depth analog-to-digital (ADC), and writing them in the resulting .bin file.
Spike sorting was performed semi-automatically using Matlab-based Kilosort 2.5 71 and resulting clusters curated in Phy2 (beta 5). Single units with <0.5% refractory period (1 ms) violations and a baseline spike rate >0.1 Hz were accepted for further analysis.

Statistical analysis of spike train data
All statistical analysis was done in Matlab 2022a or R Studio, Version 4.2.0, using built-in or custom-written functions. Unless otherwise stated, data was analyzed by two-way repeated measures ANOVA with a Bonferroni correction. See Supplementary Table 1 for exact statistical tests and test outputs (F- and p-values). Paired MI and $\bar{r}$ data across conditions (per region) were analyzed using the Friedman test, followed by a Wilcoxon signed-rank test. MI and $\bar{r}$ comparisons across regions (Supplementary Table 2) was done with a mixed-model ANOVA followed by a rank-sum test for pairwise differences. Comparisons of proportion of either positively, negatively, or unmodulated units per stimulation condition were made by a X2 proportions test, followed by the Marascuillo procedure for multiple comparisons. Statistical differences in the proportions of responsive units across conditions were assessed using McNemar's test in the case of paired data or a two-proportions X2 test in the case of unpaired data (custom written), e.g. between cortical layers.

Code is available here: https://github.com/rebecca-mease/Ziegler_et_al_2023 and releases updated at:https://zenodo.org/record/7920058#.ZFvugnbP0i4

For manuscripts utilizing custom algorithms or software that are central to the research but not yet described in published literature, software must be made available to editors and reviewers. We strongly encourage code deposition in a community repository (e.g. GitHub). See the Nature Portfolio guidelines for submitting code & software for further information.

Data

Policy information about availability of data

All manuscripts must include a data availability statement. This statement should provide the following information, where applicable:

- Accession codes, unique identifiers, or web links for publicly available datasets
- A description of any restrictions on data availability
- For clinical datasets or third party data, please ensure that the statement adheres to our policy

All the data are in the manuscript or in supplementary material. Source data are provided as a Source Data file and is available here: https://doi.org/10.11588/data/D2O0JZ.

Human research participants

Policy information about studies involving human research participants and Sex and Gender in Research.

Reporting on sex and gender: N/A

Population characteristics: N/A

Recruitment	N/A
Ethics oversight	N/A

Note that full information on the approval of the study protocol must also be provided in the manuscript.

Field-specific reporting

Please select the one below that is the best fit for your research. If you are not sure, read the appropriate sections before making your selection.

☒ Life sciences ☐ Behavioural & social sciences ☐ Ecological, evolutionary & environmental sciences

For a reference copy of the document with all sections, see nature.com/documents/nr-reporting-summary-flat.pdf

Life sciences study design

All studies must disclose on these points even when the disclosure is negative.

Sample size	Sample sizes were chosen in accordance with standard numbers of animals in comparable studies in the field, e.g. Tan et al. (2019), https://doi.org/10.1038/s41467-019-08873-z; Kirchgessner et al. (2021), https://doi.org/10.1016/j.cub.2021.09.025<br><br>The exact size of specific experimental groups are given in the figures legends and in Supplementary Table 1.
Data exclusions	No animals were excluded from the groups. No data points were excluded.
Replication	All experiments were replicated in at least five mice for the behavior and in at least three mice for the electrophysiology (except for L5-ChR2 in Fig. 6b for which n=2). Exact group numbers are given in the figure legends and in Supplementary Table 1.
Randomization	The effects described in the paper were drawn from within-animal comparisons, such that animals served as their own control and randomization would not be relevant. Randomly allocated control groups for optogenetic stimulation received stereotaxic injections of non-opsin expressing virus. Stimulus conditions in the electrophysiology part were applied interleaved to prevent non-stationary effects from anesthesia affecting the results.
Blinding	Experimenters were blinded to the experimental identity of the animals in the behavioral experiments. Stimulation and recording protocols in the electrophysiological experiments were run automatically by a computer with predefined parameters, which removes any subjective components of the data collection.

Reporting for specific materials, systems and methods

We require information from authors about some types of materials, experimental systems and methods used in many studies. Here, indicate whether each material, system or method listed is relevant to your study. If you are not sure if a list item applies to your research, read the appropriate section before selecting a response.

Materials & experimental systems

n/a	Involved in the study	
☒	☐	Antibodies
☒	☐	Eukaryotic cell lines
☒	☐	Palaeontology and archaeology
☐	☒	Animals and other organisms
☒	☐	Clinical data
☒	☐	Dual use research of concern

Methods

n/a	Involved in the study	
☒	☐	ChIP-seq
☒	☐	Flow cytometry
☒	☐	MRI-based neuroimaging

Animals and other research organisms

Policy information about studies involving animals; ARRIVE guidelines recommended for reporting animal research, and Sex and Gender in Research

Laboratory animals	Mice (male and female, 7-16 weeks of age) were housed with food and water ad libitum on a 12h light/dark cycle (housing conditions 20-22°C, 40-65% humidity).<br>The following mouse lines were used:<br>“Ntsr1-Cre” (B6.FVB(Cg)-Tg/(Ntsr1-cre)GN220Gsat/Mmucd)<br>“Ntsr1-Cre-ChR2-EYFP”; crossbreed between “Ntsr1-cre” x “Ai32” (B6.FVB(Cg)-Tg/(Ntsr1-cre)GN220Sat/Mmucd x B6.129S-Gt(ROSA)26Sortm32(CAG-COP4*H134R/EYFP).

	"Rbp4-Cre" (B6.FVB/CD1-Tg(Rbp4-cre)KL100Gsat/Mmucd)
Wild animals	No wild animals were used in this study.
Reporting on sex	Animals of both sexes were used. Sex-specific effects were not addressed in this study.
Field-collected samples	No field-collected samples were used in this study.
Ethics oversight	All experimental procedures were performed according to the ethical guidelines set by the governing body (Regierungspräsidium Karlsruhe, Germany).

Note that full information on the approval of the study protocol must also be provided in the manuscript.
